# Erosion of the Teeth

**Published:** 1895-01

**Authors:** D. E. Wiber


ARTICLE IV.

### EROSION OF THE TEETH,

BY D. E. WIBER, D. D. S.

This is a condition where a loss of enamel occurs generally at the labia-cervical portion of the teeth, usually the incisors, cuspids, and very frequently the bicuspids. The groove or depression which primarily appears, is smooth and regular, in its primary stage it is not very extensive; in some instances its ravages is slow, and again rapid, so rapid in fact that the existence of the disease is hardly known by the patient, before his condition is exceedingly grave. The color of the enamel rarely changes, but the dentine becomes pale, which is shortly followed by a dark brown color. This form of destruction to tooth substance has been termed "decay by denudation."

Erosion is found more frequently in the upper set of teeth than the lower ones. The progress of this affection is, as I have already stated, variable. When it has become fully established, and the dentine exposed, the least heat or cold. or the touch of a dental instrument, in examination, demonstrates a highly sensitive condition.

*Etiology.*—Erosion, it is claimed by some writers, is caused by chemical irritation, such as acids, etc., but to this theory there are many well grounded objections. The tooth brush has been unjustly blamed as instrumental in causing this affection; this may be flatly contradicated by pointing out the fact that erosion occurs on the lingual surfaces, and inaccessible portions of the teeth. It may, after the groove has been formed, increase its size; also it may determine the commencement of this groove, but no conceivable use of the tooth brush could cause this action. Then, again, poor quality or structure of the enamel has received some attention and support as the cause, but the most plausible reason advanced, is the action of some acid secretion, abnormal in character, produced on the inner side of the lip, the motion of the latter assisting in the solution of the tooth substance.

*Treatment.*—The most skillful treatment is necessary· Where the affection is superficial, grinding out and polishing of the eroded surface with corundum wheels is all that is demanded, and a mouth-wash prescribed. I have received flattering results lately, from the use of the milk of magnesia. This destroys the acidity of the mucous and glandular secretions, and renders the oral cavity alkaline for several hours, when its use can be repeated. When the disease is presented in an advanced stage, nothing short of excavation and filling with some durable material will arrest its advancement. Diet, in treating this disturbance is a very important factor to consider. The patient should be careful to exclude acidulous food and drink, certain fruits, and pickled condiments.—*Medical Brief.*

---

ARTICLE V.

